# Antifungal Activity of *Punica granatum* L. Peel Extract and Chitosan Nanoparticle-Coated Formulation Against *Candida* Infections

**DOI:** 10.3390/ijms27094127

**Published:** 2026-05-05

**Authors:** Kazim Sahin, Sena Sahin Aktura, Ilkay Bahceci, Derya Bal Altuntas, Muharrem Mert Otegeceli

**Affiliations:** 1 Department of Medical Microbiology, Faculty of Medicine, Recep Tayyip Erdogan University, Rize 53020, Turkey; kazim.sahin@erdogan.edu.tr (K.S.);; 2 Department of Bioengineering, Faculty of Engineering and Architecture, Recep Tayyip Erdogan University, Rize 53100, Turkey; 3 Faculty of Medicine, Recep Tayyip Erdogan University, Rize 53020, Turkey

**Keywords:** *Punica granatum*, chitosan nanoparticles, *Candida* spp., antifungal activity, MIC, MFC

## Abstract

Increasing incidence of candidiasis and emergence of antifungal resistance necessitate the development of alternative antifungal strategies. In this context, the antifungal activity of the crude extract of *Punica granatum* L. peel (PGPE) and its chitosan nanoparticle-coated formulation (PGPE-CSNPs) was investigated against *Candida albicans* (ATCC 10231), *Candida glabrata* (ATCC 66032), *Candida kefyr* (ATCC 46764), *Candida parapsilosis* (ATCC 22019), and *Candida tropicalis* (ATCC 13803). Although the individual antimicrobial activities of PGPE and CSNPs have been investigated, their combined application against *Candida* spp. remains unexplored in the literature. The antifungal efficacy was evaluated using agar well diffusion, disk diffusion, minimum inhibitory concentration (MIC), and minimum fungicidal concentration (MFC) testing, and compared with fluconazole and amphotericin B. The morphological characterization of PGPE-CSNPs was performed using scanning electron microscopy (SEM), which confirmed successful encapsulation and revealed a smoother surface with uniformly distributed nanometric pore structures and reduced aggregation compared to uncoated CSNPs. PGPE-CSNPs showed greater inhibition zones than amphotericin B, except against *C. albicans*. The CSNPs formulation reduced the MIC from 8 µg/mL to 4 µg/mL and the MFC from 16 µg/mL to 8 µg/mL, representing a two-fold enhancement against *C. albicans*. No enhancement in activity was observed against *C. glabrata*, whereas for *C. kefyr*, only MFC values decreased from 8 µg/mL to 4 µg/mL. For both *C. parapsilosis* and *C. tropicalis*, MIC values reduced from 16 µg/mL to 8 µg/mL, and MFC values reduced from 32 µg/mL to 16 µg/mL for both species. PGPE-CSNPs exhibited significantly lower MIC and MFC values than the crude extract alone (*p* < 0.05). These findings suggest that chitosan-based nanocarriers may enhance the antifungal efficacy of plant-derived bioactive compounds, highlighting their potential as a promising alternative antifungal strategy, a combinatorial approach not previously reported in the literature.

## 1. Introduction

Mycoses are fungal infections that may result in severe disease and significant treatment-related complications [1]. Among fungal pathogens, *Candida* species are considered the most clinically significant, responsible for opportunistic infections. *Candida* spp. is capable of causing life-threatening bloodstream infections, particularly in hospitalized patients with underlying conditions or invasive medical interventions [2]. Invasive fungal infections constitute an important global health problem, particularly among immunocompromised individuals and critically ill patients in intensive care units [3]. Responsible for approximately 400,000 life-threatening infections each year, invasive candidiasis is associated with high mortality rates of 29–72%, highlighting the significant clinical impact of these opportunistic pathogens [4,5].

Furthermore, antifungal-resistant *Candida* species have become a global public health concern. Recent surveillance data indicate that drug-resistant *Candida* infections increased during the COVID-19 pandemic, especially in hospital-onset cases. This highlights the growing burden of antifungal resistance in healthcare settings [6,7]. Despite the clinical importance of *Candida* infections, there are limited therapeutic options. Currently approved, three major classes of antifungal drugs are used for the treatment of systemic fungal infections: polyenes, azoles, and echinocandins [8]. These agents constitute the cornerstone of antifungal therapy for candidiasis. However, antifungal treatment options are restricted and frequently show limited efficacy in severe infections [9]. A key virulence feature of certain *Candida* species, particularly *C. albicans*, which is a major etiological agent of mucocutaneous infections, including oral and vaginal candidiasis, is the ability to undergo morphological transitions between yeast and filamentous forms [10]. The yeast form is generally associated with systemic dissemination through the bloodstream, whereas the filamentous forms facilitate adhesion, invasion, and biofilm formation during infection. This morphological plasticity plays an important role in pathogenicity, tissue invasion, and immune evasion [11]. *C. glabrata*, another common species, is notable for its reduced susceptibility to azole antifungals, particularly fluconazole. In contrast, *C. parapsilosis* is a significant cause of candidemia in neonates and immunocompromised patients, often linked to biofilm formation on indwelling medical devices. Similarly, *C. tropicalis* is characterized by high virulence and a tendency to develop fluconazole resistance. Reflecting these trends, the World Health Organization (WHO) lists *C. glabrata*, *C. parapsilosis* and *C. tropicalis* as “high priority” species due to rising multidrug resistance [12]. Furthermore, *C. kefyr* has emerged as a cause of invasive infections, especially in immunocompromised individuals and those with hematologic malignancies, and is notable for its reduced intrinsic susceptibility to echinocandins [13,14].

As a result, the increased prevalence of antifungal resistance, drug toxicity, high treatment costs, and overlapping mechanisms of action contribute to the considerably high rates of mortality and therapeutic failure [15]. In addition to antifungal resistance, antifungal tolerance has been described as a phenomenon in which a subpopulation of cells can survive exposure to antifungal agents, particularly fungistatic drugs, by slowing or halting division while retaining the ability to resume proliferation after drug removal [16]. Notably, the use of adjuvant compounds, including inhibitors of stress response pathways, has been shown to enhance the cidal activity of fluconazole and reduce tolerance, thereby more effectively eliminating tolerant cell populations [16]. Given these limitations in current antifungal therapies, there is growing interest in alternative antifungal agents derived from natural products.

Medicinal plants have long been recognized as a rich source of antimicrobial compounds. *Punica granatum* L. peel is especially rich in phenolic compounds, which have demonstrated broad antimicrobial and antifungal activity [17,18]. However, the therapeutic application of crude plant extracts is often limited by poor stability, low bioavailability, and rapid degradation [19]. Nanotechnology-based delivery systems have recently emerged as promising strategies to overcome these limitations by encapsulating plant-derived molecules within nanosized carriers. These systems prevent rapid degradation, facilitate transport across biological barriers, and increase therapeutic efficacy [19]. Chitosan nanoparticles (CSNPs) are particularly attractive due to their biocompatibility, biodegradability, and intrinsic antimicrobial activity. The cationic nature of chitosan facilitates strong electrostatic interactions with negatively charged fungal cell walls, thereby increasing membrane permeability and enhancing antifungal efficacy [20]. Although the individual antimicrobial activities of *Punica granatum* peel extract (PGPE) and chitosan nanoparticles (CSNPs) have been studied, their combined application against *Candida* spp. has not been investigated. Despite the increasing number of studies on plant-derived antifungal compounds, clinical translation remains limited, underscoring the need for improved delivery systems that enhance stability and bioavailability. Therefore, we hypothesized that encapsulating PGPE in chitosan nanoparticles (PGPE-CSNPs) would enhance antifungal activity against clinically relevant *Candida* species compared with the crude extract and achieve efficacy similar to that of standard antifungal agents. To test this hypothesis, we employed agar well diffusion, disk diffusion, minimum inhibitory concentration (MIC), and minimum fungicidal concentration (MFC) assays.

## 2. Results

### 2.1. Chemical Composition of PGPE

Phenolic compounds of PGPE were identified and quantified, as shown in Table 1, that component listed in descending order of concentration. Punicalagin isomers, punicalagin A and punicalagin B, exhibited the highest concentrations at 4528.03 µg/g and 2591.46 µg/g. Epigallocatechin gallate (EGCG) was another abundant compound (186.88 µg/g), while chlorogenic acid (60.39 µg/g) and ellagic acid (46.38 µg/g) were present in substantial amounts. Other phenolic acids and flavonoids, including caffeine, 2,4-dihydroxybenzoic acid, catechin hydrate, ferulic acid, gallic acid, ursolic acid, caffeic acid, quercetin, and p-coumaric acid, were detected at lower concentrations ranging from 16.32 µg/g to 0.19 µg/g.

### 2.2. Chitosan Characterization Analysis of PGPE-CSNPs

#### 2.2.1. Zeta Potential

**Figure 1:**
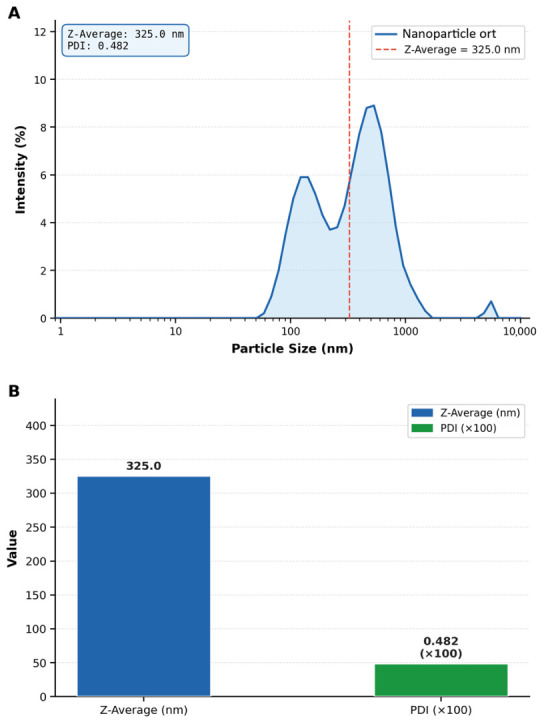
Zetasizer Nano ZSP characterization of NPs. (**A**) Intensity-weighted particle size distribution showing Z-Average = 325.0 nm and PDI = 0.482. The dashed red line indicates the Z-Average position. (**B**) Summary bar chart of key physicochemical parameters.

The Z-average of ~325 nm with a PDI value of 0.482 indicated acceptable size distribution homogeneity (Figure 1). In this context, particle sizes in the 300–500 nm range have been reported to be effective for topical antifungal delivery [21,22,23]. As expected for hydrophilic polymeric nanoparticles, the hydrodynamic Z-mean (325.0 nm) measured by Zetasizer Nano ZSP instrument is larger than the particle sizes observed by SEM imaging the anhydrous particles under vacuum [24].

The high positive zeta potential (+47.7 mV) of PGPE-CSNPs promotes strong electrostatic attraction with the negatively charged cell wall of *Candida* species. This interaction, rather than cellular internalization, is the primary antifungal mechanism. Supporting this, our in vitro MIC results demonstrate significantly greater antifungal activity of PGPE-CSNPs than crude PGPE across all tested *Candida* species (*p* < 0.05), validating the functional efficacy of the formulation at this size.

#### 2.2.2. Scanning Electron Microscopy (SEM)

**Figure 2:**
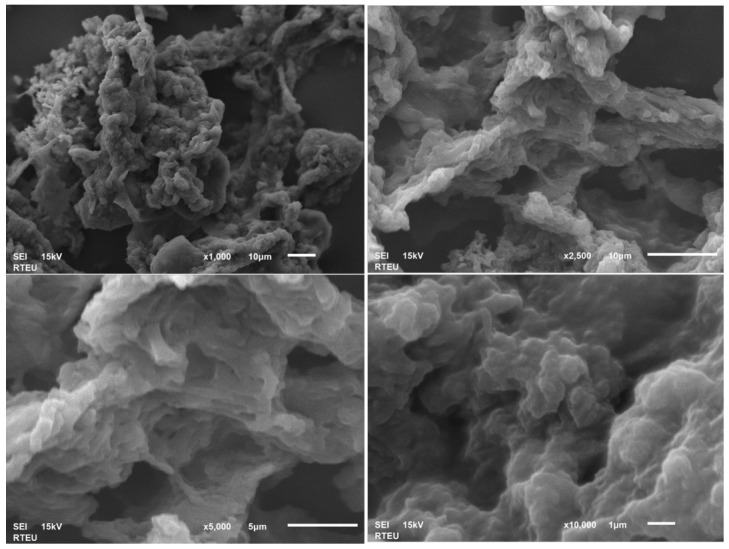
SEM images of CSNPs at magnifications of ×1000, ×2500, ×5000, and ×10,000. Scale bars represent 10 µm (×1000 and ×2500), 5 µm (×5000), and 1 µm (×10,000). SEI mode, 15 Kv.

**Figure 3:**
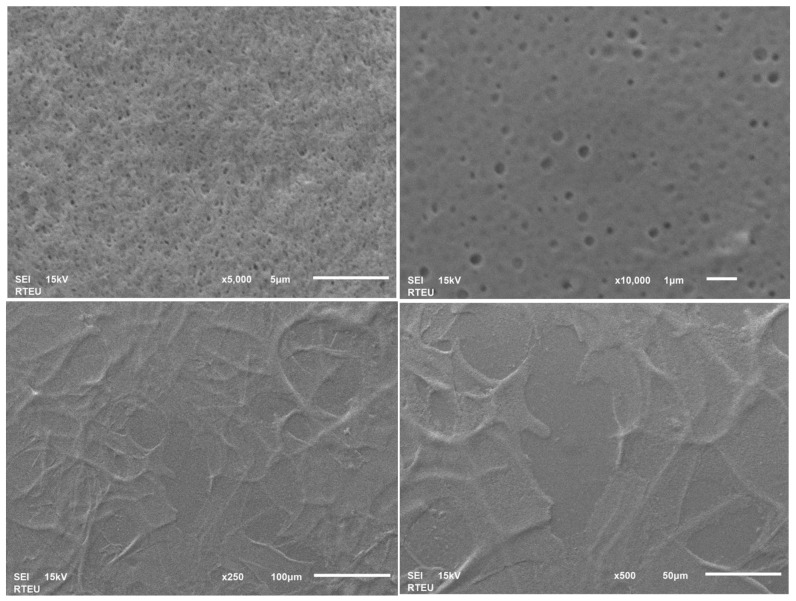
SEM images of PGPE- CSNPs at magnifications of ×250, ×500, ×5000, and ×10,000. Scale bars represent 100 µm (×250), 50 µm (×500), 1 µm (×10,000) and 5 µm (×5000). SEI mode, 15 Kv.

The CSNP and PGPE-CSNP samples were lyophilized and gold-coated prior to scanning electron microscopy (SEM) imaging. For CSNPs, images at four magnifications (×1000, ×2500, ×5000, and ×10,000) are shown in Figure 2, whereas for PGPE-CSNPs, images at ×250, ×500, ×5000, and ×10,000 are shown in Figure 3.

SEM analysis revealed striking morphological differences between the two formulations: CSNPs exhibited an irregular, rough, and fibrous network-like microstructure with prominent aggregates, characteristic of the ionic gelation process, whereas PGPE-CSNPs displayed a dramatically transformed surface that was markedly smoother, more compact, and highly homogeneous, with uniformly distributed nanometric pore structures visible at ×5000 and ×10,000 magnifications, interpreted as evidence of phenolic PGPE compounds, particularly punicalagin, adsorbed onto the chitosan matrix. The flat, sheet-like aggregate formations observed at lower magnifications (×250−×500) further indicate that PGPE comprehensively enveloped the nanoparticle surface during the coating process. Taken together with the high positive zeta potential (+47.7 mV) and data (Z-average: 325.0 nm; PDI: 0.482), these morphological observations collectively confirm that PGPE was successfully encapsulated and that surface modification of the chitosan nanoparticles was effectively achieved.

SEM analysis revealed distinct morphological differences between CSNPs and PGPE-CSNPs, providing direct visual evidence of successful PGPE encapsulation as summarized in Table 2.

CSNPs exhibited an irregular, rough, fibrous-network surface with prominent aggregation, large and irregular open pores, and a wrinkled layered macrostructure. In contrast, PGPE-CSNPs displayed a smooth, compact, homogeneous surface with reduced aggregation, uniformly distributed nanometric pore dots, and flat, broad sheet-like structures, confirming the successful encapsulation of PGPE onto the chitosan nanoparticles.

### 2.3. Agar Well Diffusion Analysis

**Figure 4:**
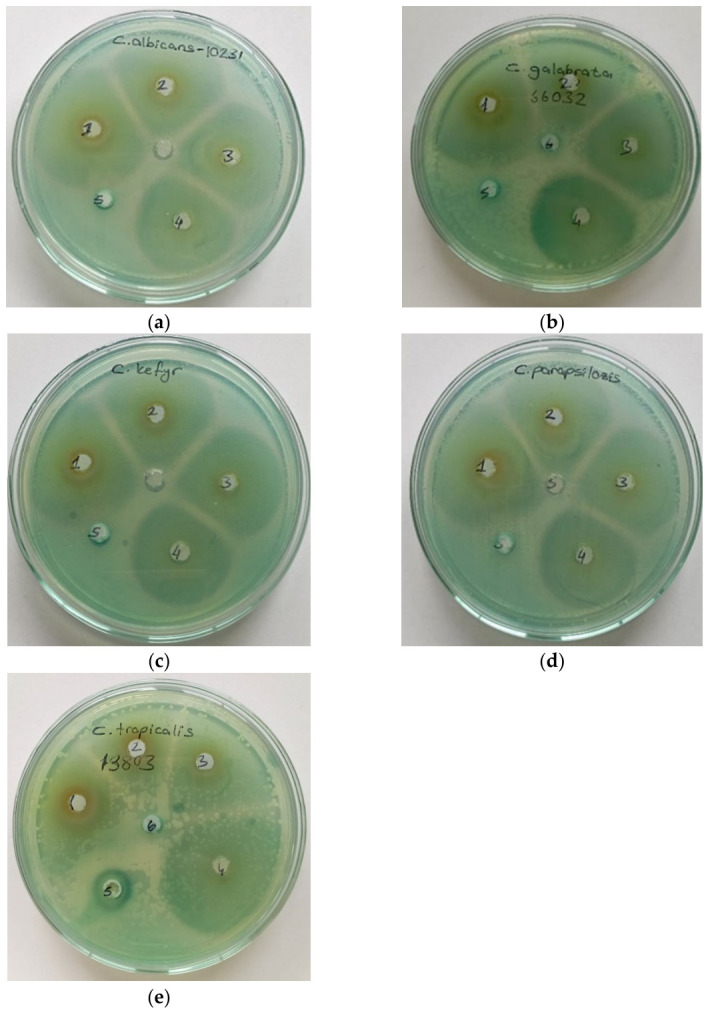
Inhibition zones created by PGPE against *Candida* spp. (**a**) *C. albicans* ATCC 10231, (**b**) *C. glabrata* ATCC 66032, (**c**) *C. kefyr* ATCC 46764, (**d**) *C. parapsilosis* ATCC 22019, (**e**) *C. tropicalis* ATCC 13803 (1: 800 µg/mL, 2: 400 µg/mL, 3: 200 µg/mL, 4: 100 µg/mL, 5: 50 µg/mL, 6: negative control).

The antifungal activity of PGPE against at concentrations ranging from 50 to 800 µg/mL and inhibition zone diameters (mean ± SD) are presented in Figure 4 and Table 3. For *C. albicans*, inhibition zones ranged from 25.00 ± 1.00 mm (200 μg/mL) to 28.33 ± 2.89 mm (800 μg/mL), with no clear dose-dependent trend. *C. glabrata* showed increasing inhibition with higher concentrations, from 13.67 ± 1.53 mm at 50 μg/mL to 29.33 ± 3.79 mm at 800 μg/mL. *C. kefyr* exhibited the highest susceptibility to PGPE at all concentrations. *C. parapsilosis* displayed the smallest inhibition zones among all concentrations tested, ranging from 15.67 ± 7.51 mm (50 μg/mL) to 26.67 ± 5.77 mm (800 μg/mL). *C. tropicalis* showed relatively stable inhibition across all concentrations, with diameters between 23.67 ± 0.58 mm and 27.33 ± 2.31 mm, and no growth inhibition was observed in the negative control for any species. The results demonstrated that *C. kefyr* was the most susceptible, followed by strong and consistent activity against *C. albicans* and *C. tropicalis*; *C. glabrata* showed moderate susceptibility, while *C. parapsilosis* was the least susceptible. PGPE exhibited significant in vitro antifungal activity against all tested *Candida* species, and the effect was concentration-dependent and varied among the tested species.

### 2.4. Disk Diffusion Assay

The inhibition zone diameters of PGPE, PGPE-CSNPs, amphotericin B (AMB), and fluconazole (FLU) are given in Table 4.

Against *C. albicans*, FLU produced the largest inhibition zone (32.33 ± 2.08 mm), followed by PGPE (28.33 ± 2.89 mm), while PGPE-CSNPs (15.67 ± 0.58 mm) and AMB (17.67 ± 0.58 mm) showed smaller zones. For *C. glabrata*, PGPE-CSNPs exhibited the largest inhibition zone (35.67 ± 1.15 mm), which was significantly larger than AMB (16.33 ± 0.58 mm, *p* = 0.001), FLU (29.00 ± 1.73 mm, *p* = 0.043), and PGPE (29.33 ± 3.79 mm, *p* = 0.027). In *C. kefyr*, FLU gave the highest activity (43.33 ± 5.77 mm), followed by PGPE-CSNPs (37.67 ± 0.58 mm) and PGPE (31.67 ± 0.58 mm), while AMB had the smallest zone (12.33 ± 0.58 mm). All pairwise comparisons involving AMB and FLU or PGPE were significant (*p* < 0.01). For *C. parapsilosis*, FLU also produced the largest zone (36.00 ± 3.46 mm), significantly larger than AMB (16.00 ± 0.00 mm, *p* < 0.001), PGPE (26.67 ± 5.77 mm, *p* = 0.014), and PGPE-CSNPs (29.00 ± 1.00 mm, *p* = 0.022). Both PGPE and PGPE-CSNPs showed significantly higher antifungal activity than AMB (*p* = 0.016 and *p* = 0.003, respectively). Against *C. tropicalis*, FLU showed the largest zone (34.00 ± 2.65 mm), followed by PGPE-CSNPs (29.00 ± 1.00 mm) and PGPE (27.33 ± 2.31 mm), whereas AMB had the smallest (16.00 ± 0.00 mm); however, no statistical comparisons were provided for this species. The results suggest that FLU showed the largest inhibition zones in all other groups except *C. glabrata*. PGPE and PGPE-CSNPs also performed significantly better than AMB, which had the smallest inhibition zones against *C. glabrata*, *C. kefyr*, *C. parapsilosis*, and *C. tropicalis*.

### 2.5. MIC and MFC Tests

**Figure 5:**
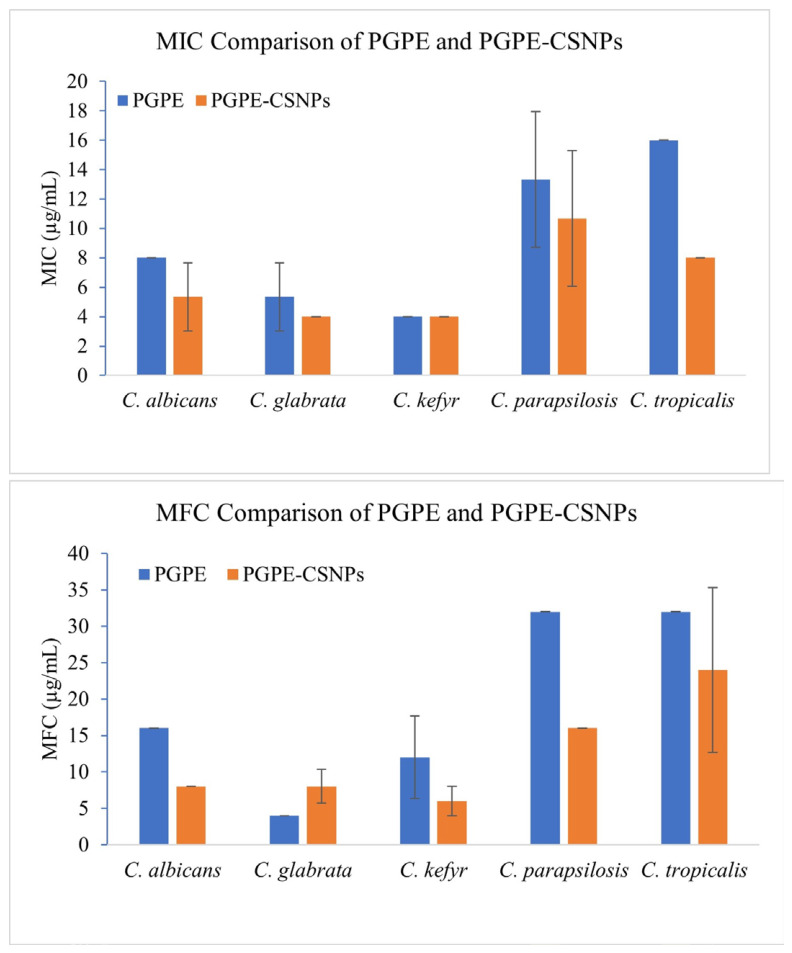
Comparison of MIC and MFC values between PGPE and PGPE-CSNPs.

The MIC and MFC values of PGPE and PGPE-CSNPs against five *Candida* species are presented in Figure 5 and Table 5. The nanoformulation exhibited enhanced antifungal activity compared to the crude extract, as evidenced by lower MIC and MFC values for most tested strains. Against *C. albicans*, PGPE demonstrated a MIC value of 8 µg/mL, and was reduced to 4 in PGPE-CSNPs. Similarly, the MFC value of 16 µg/mL was reduced to 8 µg/mL, indicating a two-fold increase in antifungal efficacy. PGPE and PGPE-CSNPs showed identical MIC and MFC values of 4 µg/mL, suggesting that PGPE-CSNPs did not further improve activity against *C. glabrata*. Notably, PGPE-CSNPs exhibited enhanced fungicidal activity against *C. kefyr*, reducing the MFC from 8 µg/mL (PGPE) to 4 µg/mL, while the MIC remained constant at 4 µg/mL. The most pronounced effects were also observed against *C. parapsilosis* and *C. tropicalis*, where both MIC and MFC values decreased by two-fold following nanoencapsulation. For these species, PGPE alone yielded MIC value of 16 µg/mL, whereas PGPE-CSNPs reduced these to 8 µg/mL and MFC value 32 µg/mL, reduced to 16 µg/mL. Overall, the chitosan nanoparticle formulation enhanced the antifungal activity of PGPE, with the most substantial improvements observed against *C. albicans*, *C. parapsilosis*, and *C. tropicalis*.

The nanoparticle-coated formulation exhibited lower MIC and MFC values against most tested *Candida* spp., indicating stronger antifungal activity. An MFC/MIC ratio of ≤4 was interpreted as fungicidal activity, whereas a ratio > 4 indicated fungistatic activity [25]. The crude PGPE extract demonstrated fungicidal activity against all five *Candida* species, including *C. albicans*, *C. glabrata*, *C. kefyr*, *C. parapsilosis*, and *C. tropicalis*, with MFC/MIC ratios ranging from 1 to 2. Similarly, PGPE-CSNPs also exhibited fungicidal activity against all tested *Candida* species, maintaining ratios ≤ 4.

## 3. Discussion

*Candida* spp. is the most common fungal pathogen, with *C. albicans* being the most prevalent species (49.36%), followed by *C. tropicalis* (21.89%), *C. parapsilosis* (13.92%), and *C. glabrata* (11.37%). Susceptibility testing reveals that the lowest susceptibilities are observed for the azole class; for instance, fluconazole susceptibility rates are 93.25% for *C. parapsilosis*, 91.6% for *C. albicans*, 79.4% for *C. glabrata*, 77.95% for *C. tropicalis*. Conversely, amphotericin B and anidulafungin demonstrate the highest efficacy across all tested *Candida* species [26]. In the other study, similarly, *C. albicans* remains the predominant pathogen, while *C. glabrata* has emerged as the second most common cause of invasive candidiasis, alongside several less common species capable of developing resistance to triazoles and amphotericin B [27]. Furthermore, increasing reports indicate that clinical isolates are resistant to azoles, echinocandins, and polyenes; *C. glabrata* remains a major concern for acquired resistance [28]. *C. albicans* is the most susceptible species to nearly all antifungal classes. Nivoix et al. report that fluconazole exhibits good activity against *C. albicans*, with MIC values typically within the susceptible range. Similarly, amphotericin B also shows potent activity. Among non-albicans *Candida* species, *C. glabrata* shows poor/absent activity to fluconazole, whereas amphotericin B retains moderate activity. *C. parapsilosis*, fluconazole, and amphotericin B are highly active. *C. tropicalis*, conversely, is uniformly susceptible to both antifungal classes [29]. An effective antifungal strategy against *Candida* infections should integrate the pharmacological distinction between fungistatic and fungicidal agents. Antifungal resistance among *Candida* species represents a significant clinical challenge, largely due to the limited availability of antifungal drug classes. Widespread azole resistance constitutes a major barrier to effective treatment. Additionally, the increasing prevalence of echinocandin and multidrug resistance, particularly in *C. glabrata*, further complicates treatment [15]. In contrast, resistance to polyenes, such as amphotericin B, is infrequent and rarely observed in clinical settings [30]. Nevertheless, the clinical utility of polyenes is constrained by dose-dependent adverse effects, especially nephrotoxicity [31]. Therefore, the development of novel antifungal formulations is highlighted as a critical priority by current epidemiological trends and therapeutic gaps [32]. The peel of *Punica granatum* L. (pomegranate) is notably rich in phenolic compounds, which possess broad-spectrum antimicrobial activity [33,34]. Several studies have demonstrated its efficacy against a range of pathogens, including dermatophytes and *Candida* species [35,36,37]. Our previous studies also showed, therapeutic efficacy against sepsis, the antimicrobial potential, and protective effects of *P. granatum* [38,39,40]. Despite promising findings, the therapeutic application of crude plant extracts is frequently limited by low stability, poor solubility, and restricted bioavailability [19].

In this context, nanotechnology-based delivery systems have emerged as transformative tools in modern antifungal therapy. Among these, chitosan nanoparticles (CSNPs) stand out, offering significant advantages over conventional formulations by improving drug solubility, stability, and targeted delivery [41,42]. Chitosan, a natural aminopolysaccharide derived from deacetylated chitin, possesses antioxidant, antimicrobial, and fungicidal activities. Its small size, high surface charge, and stability make CSNPs highly attractive for biomedical applications, enabling efficient drug loading and controlled release, ultimately improving therapeutic outcomes [42,43,44]. The antifungal activity is primarily attributed to the cationic structure of chitosan nanoparticles. The amino groups of CSNPs, carrying a positive charge, interact with anionic components of the fungal cell wall, leading to membrane disruption [41]. The inhibitory effect of CSNPs on fungal membranes has been demonstrated using propidium iodide, a fluorescent dye that assesses nucleic acids and cell membrane integrity [41]. Importantly, CSNPs exhibit potent fungicidal activity against resilient biofilm-embedded forms of various *Candida* species. The ability to form biofilms constitutes a key virulence trait in persistent infections and device-related candidiasis [44,45]. CSNP-based nanocomposites can completely suppress hyphal formation and biofilm development in *C. albicans*, thereby targeting major virulence determinants [44]. Additionally, CSNPs exhibit enhanced antifungal efficacy by downregulating critical resistance genes including SNQ2 and ERG11 in *C. glabrata* and CDR1 and MDR1 in *C. tropicalis* [42]. Aghaei et al. demonstrated successful encapsulation of ribavirin, carboplatin, and *Thymus vulgaris* essential oil by chitosan nanoparticles. The encapsulation efficiencies ranged from 81 to 91%, the particle sizes were 286−298 nm, and the zeta potentials were +33 to +34 mV [42]. Encapsulated drugs showed higher minimum inhibitory concentration (MIC) values against *C. glabrata* and *C. tropicalis.* Chitosan nanoparticles loaded with carvacrol also exhibited strong activity against *C. tropicalis* [45]. *Thymus daenensis* essential oil loaded into chitosan nanoparticles formed spherical particles, averaged 92.3 nm in size, had a zeta potential of +48.3 mV, and an encapsulation efficiency of 88.6%. Capsulation significantly reduced BCR1 gene expression in *C. parapsilosis* isolates [43].

Numerous extracts and plant-derived polyphenolic compounds have demonstrated antifungal activity against a range of *Candida* species, most notably *C. albicans*, with low MIC and notable inhibition zones [46]. Similarly, *P. granatum* extract (PGPE) showed efficacy against several *Candida* species, including the resistant *C. glabrata*. Furthermore, encapsulation of PGPE resulted in lower MIC and MFC values and larger inhibition zone diameters than the non-encapsulated extract, indicating enhanced antifungal activity. MIC is defined as the lowest concentration that inhibits visible growth of the microorganism, and MFC is the lowest concentration that kills fungal cells. MIC determination is widely used to evaluate antifungal efficacy and compare the activity of natural products with that of conventional antifungal agents [47]. Nayak et al. demonstrated that encapsulating chitosan within phytic acid−crosslinked chitosan nanoparticles significantly improved antifungal activity against *C. albicans*. The encapsulation process yielded nanoparticles with a hydrodynamic diameter of 103 ± 3.9 nm, a polydispersity index (PDI) of 0.33, and a zeta potential of 37 ± 2.5 mV. The encapsulated nanoparticles exhibited MIC of 140 ± 2.2 µg/mL against *C. albicans* [48]. These findings corroborate our results showing that encapsulation reduces MIC and MFC values compared to non-encapsulated extracts. In our study, PGPE-CSNPs exhibited a Z-average of 325.0 nm with a PDI of 0.482. Encapsulation consistently lowered MIC and MFC values against *C. albicans*, *C. glabrata*, *C. kefyr*, *C. parapsilosis*, and *C. tropicalis*. Furthermore, for most tested strains, PGPE-CSNPs produced larger inhibition zone diameters compared to PGPE. These observations are consistent with our study, where encapsulation enhanced inhibition zone diameters. The reductions in MIC and MFC values following encapsulation, together with larger inhibition zones, strongly support the notion that nanoencapsulation potentiates the antifungal efficacy of natural compounds. Chitosan encapsulated pomegranate demonstrated the antifungal activity against *Candida* spp. Endo et al. showed that *C. albicans* ATCC 10231 had MIC and MFC values of 3.9 µg/mL, against *C. tropicalis* ATCC 28707; the MIC and MFC were 3.9 µg/mL and > 250 µg/mL. *C. parapsilosis* ATCC 22019: the MIC was 3.9 µg/mL, while the MFC exceeded 250 µg/mL. On the other hand, extract-loaded microparticles exhibited an MIC value of 15.6 µg/mL against *C. albicans*. MIC values of extract-loaded microparticles were found to have a higher antifungal effect than that of the free extract [49]. In addition, encapsulated clove essential oil has been demonstrated to exhibit superior antifungal activity against *Aspergillus niger* isolated from spoiled pomegranate compared to free oil and blank chitosan nanoparticles [50]. This finding supports the strategy of encapsulating plant extracts with chitosan-based nanoparticles to enhance antimicrobial efficacy. Salem et al. demonstrate the successful biosynthesis of selenium nanoparticles using pomegranate peel extract and chitosan nanoparticles. While chitosan nanoparticles alone reduced green mold lesions by 91.7%, incorporating pomegranate peel extract into the chitosan matrix increased the reduction rate to 95.4%, indicating a synergistic effect [51]. Alotaibi and colleagues observed that chitosan-based edible films, particularly incorporating pomegranate peel extract, demonstrated potent antifungal activity. This finding corroborates previous studies demonstrating that chitosan nanocomposites incorporating plant extracts can provide effective protection against a broad spectrum of fungal pathogens [52]. Furthermore, nanoparticle systems can improve the solubility and stability of plant-derived phenolic compounds, thereby enhancing their antifungal effectiveness.

In the study, the relatively close proximity between MIC and MFC values suggests that *P. granatum* peel extract exhibits predominantly fungicidal rather than fungistatic activity against several *Candida* species (MFC/MIC ratio ≤ 4). Fungicidal activity is considered particularly desirable in the management of invasive candidiasis, especially in immunocompromised patients, where complete eradication of fungal pathogens is required for successful therapy [29]. The fungicidal profile was more pronounced in the nanoparticle-coated formulation, as PGPE-CSNPs showed lower MFC values and greater fungicidal activity against all tested species. In this context, the enhanced fungicidal activity observed for PGPE-CSNPs suggests that nanoparticle-based delivery may improve the antifungal efficacy of plant-derived compounds.

Nevertheless, several limitations of the study include that all experiments were conducted in vitro, which may not directly translate into in vivo efficacy. Host immune responses, serum protein interactions, and nanoparticle biodistribution were not evaluated. While PGPE-CSNPs exhibited MIC and MFC values comparable to fluconazole and, in some cases, approaching those of amphotericin B, conventional antifungals possess well-established pharmacokinetic properties and extensive clinical validation. Therefore, the nanoparticle formulation should be considered as a potential complementary strategy rather than a direct replacement for existing antifungal therapies.

Overall, the findings of this study support the growing body of evidence that nanoparticle-based delivery systems can enhance the antifungal activity of plant-derived compounds. Chitosan-coated *P. granatum* peel extract represents a promising candidate for further investigation as a potential adjunct antifungal approach, particularly against *Candida* species exhibiting reduced susceptibility to conventional antifungal agents.

## 4. Materials and Methods

### 4.1. Preparation of Punica Granatum Peel Extract (PGPE)

*P. granatum* L. was obtained from local bazaar in Adıyaman, Türkiye. 15 g of dried peels of *P. granatum* were suspended in 100 mL of ethanol (EtOH) (Merck GmbH, Darmstadt, Germany)–water (70:30, *v*/*v*) and extracted on IKA KS 4000 i control rotary shaker (IKA-Werke GmbH & Co. KG, Staufen, Germany) at room temperature for 48 h. The extract was filtered through Whatman No.1 filter paper (Cat. No. 1001-125; GE Healthcare, Maidstone, UK) and evaporated at 40 °C using a rotary evaporator (LabTech EV311; Labtech S.r.l., Sorisole, Italy). The obtained residue was resuspended in distilled water to obtain a stock solution of 100 mg/mL and stored at 4 °C. Test concentrations were prepared by diluting the stock solution with distilled water, which was also used as the negative control.

### 4.2. Phytochemical Analysis of PGPE

The chemical composition of the PGPE was analyzed using high-performance liquid chromatography (HPLC) system (Shimadzu LC-2030C-3D; Shimadzu Corporation, Kyoto, Japan) equipped with Shimadzu LabSolutions software (version 5.93; Shimadzu Corporation, Kyoto, Japan) as described previously by Sahin Aktura et al. and the extraction was performed under optimized conditions using 50% methanol (Merck GmbH, Darmstadt, Germany), which was previously identified as the most effective solvent for recovering bioactive compounds [40]. The identification and quantification of the major compounds were carried out by comparing the retention times and peak areas with those of reference standards.

### 4.3. Preparation of Chitosan Nanoparticles, Characterization and Extract Loading

**Figure 6:**
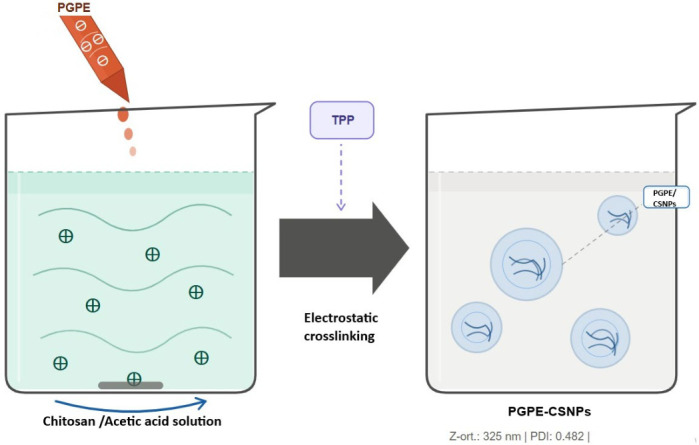
Illustration of the synthesis steps of nanoparticles.

Chitosan nanoparticles were prepared using the ionic gelation method [53]. Chitosan (Cat. No. C2395; TCI Chemicals, Zwijndrecht, Belgium) was dissolved in 1% (*v*/*v*) acetic acid (Merck KGaA, Darmstadt, Germany) solution under magnetic stirring to obtain a clear solution. Sodium tripolyphosphate (TPP; Cat. No. P0272; TCI Chemicals, Zwijndrecht, Belgium) was used as a cross-linking agent. Nanoparticle formation was achieved by the dropwise addition of TPP solution into the chitosan solution under constant stirring at room temperature. PGPE was incorporated into the chitosan nanoparticle system during the nanoparticle formation process to obtain extract-loaded chitosan nanoparticles (PGPE-CSNPs). An illustration of the synthesis steps of nanoparticles is shown in Figure 6.

The physicochemical characterization of PGPE-CSNPs, including particle size, polydispersity index (PDI), and zeta potential, was performed as described in our previous study [38].

The synthesized nanoparticles were characterized with a Zetasizer Nano ZSP instrument (Malvern Panalytical, Malvern, UK). This technique simultaneously provided the hydrodynamic particle size (Z-average), PDI, and zeta potential through electrophoretic light scattering (ELS). Measurements were performed in triplicate at 25 °C. Additionally, morphological characterization was performed by Scanning Electron Microscopy (SEM) using a JEOL JSM-IT200 instrument (JEOL Ltd., Tokyo, Japan) and JEOL 6610 scanning electron microscope (JEOL Ltd., Tokyo, Japan) at 15 kV accelerating voltage in Recep Tayyip Erdogan University (RTEU). The CSNP and PGPE-CSNP samples were lyophilized and coated with gold prior to imaging.

### 4.4. Test Strains and Culture Conditions

Antifungal activity was evaluated against five yeast strains, *Candida albicans* (ATCC 10231), *C. glabrata* (ATCC 66032), *C. kefyr* (ATCC 46764), *C. parapsilosis* (ATCC 22019), and *C. tropicalis* (ATCC 13803). All strains were cultured on Sabouraud Dextrose Agar (SDA) for maintenance and subsequent inoculum preparation. For the antifungal susceptibility testing, Mueller-Hinton Agar supplemented with 2% glucose and methylene blue (MH-GMB) was used as the assay medium to enhance the visualization and definition of inhibition zones of *Candida* species. Prior to testing, fungal suspensions were prepared from 24 h fresh cultures and adjusted to a standardized inoculum density of 0.5–2.5 × 10^3^ CFU/mL.

### 4.5. Antifungal Susceptibility Testing

#### 4.5.1. Agar Well Diffusion Method

The antimicrobial activity of PGPE at concentrations ranging from 50 to 800 µg/mL, was evaluated using the agar well diffusion method. 6 mm wells were opened in the *Candida* spp. inoculated MH-GMB agar, and 50 μL of each extract was introduced into the wells [54]. Distilled water was used as a negative control. The plates were then incubated at 35 °C for 48 h. The antimicrobial activity was determined by measuring the diameter of the inhibition zones (mm) around the wells.

#### 4.5.2. Disk Diffusion Method

Dry and sterile disks were impregnated with 10 μL of PGPE-CSNPs (800 µg/mL) and applied to the yeast suspension inoculated agar surface. Fluconazole (25 µg) and amphotericin B (100 µg) disks were used as reference antifungal controls. Plates were incubated at 35 °C for 48 h, and antifungal activity was assessed by measuring the diameters of the inhibition zones (mm) around each disk.

#### 4.5.3. Determination of Minimum Inhibitory Concentration (MIC) and Minimum Fungicidal Concentration (MFC) Values

Minimum inhibitory concentration (MIC) values were determined using the broth microdilution method according to the EUCAST guidelines, using RPMI 1640 as the growth medium [55]. Serial two-fold dilutions of the tested samples were prepared in 96-well microtiter plates. After inoculation, the plates were incubated at 35 °C for 48 h. The MIC was determined as the lowest concentration that resulted in a significant reduction in absorbance, indicating inhibition of fungal growth compared to growth control.

Minimum fungicidal concentration (MFC) values were determined by subculturing 10 μL aliquots from the MIC and higher concentration wells onto SDA. After incubation at 35 °C for 24 h, the lowest concentration resulting in no colony growth (≥99.9% killing) was recorded as the MFC. The fungicidal or fungistatic activity of the samples was determined based on the MFC/MIC ratio, where a ratio ≤ 4 was considered fungicidal and > 4 was considered fungistatic [25].

The anticandidal activity of PGPE was evaluated using the microdilution method, with two-fold serial dilutions ranging from 512 to 2 µg/mL. Fluconazole was used as a positive control. Following incubation, the optical density of each well was measured at 600 nm using a spectrophotometer.

### 4.6. Statistical Analysis

All experiments were performed in triplicate. Data are presented as mean ± standard deviation (SD). Statistical differences between groups were analyzed using one-way analysis of variance (ANOVA) followed by Tukey’s multiple comparison test. A *p*-value < 0.05 was considered statistically significant.

## 5. Conclusions

In conclusion, *P. granatum* peel extract exhibits significant antifungal activity against *C. albicans*, *C. parapsilosis*, and *C. tropicalis*, which is markedly enhanced through chitosan nanoparticle formulation. The MIC and MFC findings indicate that PGPE-CSNPs represent a promising natural antifungal candidate, particularly with improved fungicidal activity observed against *C. albicans, C. kefyr*, *C. parapsilosis,* and *C. tropicalis*. However, neither formulation showed activity against *C. glabrata* under the tested conditions. Further in vivo studies and toxicity assessments are recommended to support future clinical applications.

## Figures and Tables

**Table 1:** Phenolic Compounds Identified in PGPE.

Compound	Concentration (µg/g)
Punicalagin (PUN) (A)	4528.03
Punicalagin (PUN) (B)	2591.46
Epigallocatechingallate (EGCG)	186.88
Chlorogenicacid (CGA)	60.39
Ellagic acid (EA)	46.38
Caffeine (CAF)	16.32
2,4-dihydroxybenzoicacid (2,4-DHBA)	11.19
Catechinhydrate (CH)	5.67
Ferulic acid (FA)	4.42
Gallic acid (GA)	2.89
Ursolicacid (UA)	2.44
Caffeic acid (CA)	1.58
Quercetin (Q)	0.78
p-coumaric acid (p-CA)	0.19

**Table 2:** Comparative SEM morphological analysis of CSNPs and PGPE-CSNPs.

Parameter	CSNPs	PGPE-CSNPs
Surface morphology (SEM observation)	Irregular, rough, fibrous-network structure	Smooth, compact, homogeneous surface
Aggregation level (particle clustering)	Prominent aggregate formation present	Reduced aggregation−PGPE coating effect
Surface pore structure	Large, irregular, open pores	Nanometric, uniform pore dots (PGPE)
Macroscale structure	Wrinkled, layered network form	Flat, broad sheets−PGPE coating layers

Observations were obtained using a JEOL 6610 scanning electron microscope at 15 kV accelerating voltage. Magnification levels of ×1000, ×2500, ×5000, and ×10,000 were applied for CSNPs; ×250, ×500, ×5000, and ×10,000 for PGPE-CSNPs.

**Table 3:** Inhibition zone diameters of PGPE (mean ± SD).

	PGPE (µg/mL)					
	800	400	200	100	50	NC
*C. albicans*	28.33 ± 2.89	27.33 ± 2.31	25.00 ± 1.00	27.33 ± 2.52	25.67 ± 2.08	0
*C. glabrata*	29.33 ± 3.79	24.00 ± 2.00	22.67 ± 2.31	21.33 ± 1.15	13.67 ± 1.53	0
*C. kefyr*	31.67 ± 0.58	30.33 ± 0.58	30.00 ± 0.00	30.00 ± 0.00	28.00 ± 0.00	0
*C. parapsilosis*	26.67 ± 5.77	22.33 ± 6.03	20.00 ± 6.93	17.33 ± 6.35	15.67 ± 7.51	0
*C. tropicalis*	27.33 ± 2.31	26.33 ± 0.58	23.67 ± 0.58	23.67 ± 2.89	23.67 ± 2.89	0

NC: negative control, distilled water.

**Table 4:** Antifungal susceptibility profile of *Candida* spp. expressed as mean inhibition zones (mm).

	PGPE	PGPE-CSNPs	AMB	FLU
*C. albicans*	28.33 ± 2.89	15.67 ± 0.58	17.67 ± 0.58	32.33 ± 2.08 ^a,b,c^
*C. glabrata*	29.33 ± 3.79 ^a,b^	35.67 ± 1.15 ^a,b^	16.33 ± 0.58	29.00 ± 1.73 ^a^
*C. kefyr*	31.67 ± 0.58 ^a^	37.67 ± 0.58 ^a^	12.33 ± 0.58	43.33 ± 5.77 ^a,b,c^
*C. parapsilosis*	26.67 ± 5.77 ^a^	29.00 ± 1.00 ^a,b^	16.00 ± 0.00	36.00 ± 3.46 ^a,b,c^
*C. tropicalis*	27.33 ± 2.31 ^a^	29.00 ± 1.00 ^a,b^	16.00 ± 0.00	34.00 ± 2.65 ^a,b,c^

All experiments were performed in triplicate, and the results are expressed as mean ± standard deviation (SD). One-Way ANOVA/Tukey HSD. ^a^ *p* < 0.05: vs. AMB, ^b^ *p* < 0.05: vs. PGPE, ^c^ *p* < 0.05: vs. PGPE-CSNPs.

**Table 5:** MIC and MFC Values of PGPE and PGPE-CSNPs (µg/mL).

	PGPE		PGPE-CSNPs	
	MIC (µg/mL)	MFC (µg/mL)	MIC (µg/mL)	MFC (µg/mL)
*C. albicans*	8	16	4	8
*C. glabrata*	4	4	4	4
*C. kefyr*	4	8	4	4
*C. parapsilosis*	16	32	8	16
*C. tropicalis*	16	32	8	16

PGPE: *Punica granatum* peel extract; PGPE-CSNPs: chitosan nanoparticle-coated PGPE; MIC: minimum inhibitory concentration; MFC: minimum fungicidal concentration.

## Data Availability

The data presented in this study will be available upon request to the corresponding author.
